# Report of Deaths in the City of Buffalo for the Month of October, 1861

**Published:** 1861-12

**Authors:** J. Whittaker

**Affiliations:** Health Physician


*Report of Deaths in the City of Buffalo for the month of October,* 1861

Accidents 10, Anxmia 1, Angina Maligna 3, Apoplexy of Lungs 1, Aptha 1, Cancer of Stomach 1, Cholera Infantum 13, Consumption 16, Convulsions 12, Croup 2, Debility 8, Dentition 1, Diabetes Mellitus 1, Diarrhœa 21, Disease of the Brain 1, Disease of the Heart 3, Disease of the Lungs 2, Disease of the Spine 1, Diptheria 2, Dropsy 1, Dropsy of the Brain 2, Dysentery 10, Empyema 1, Fever Remittent 1, Fever Scarlet 5, Fever Typhoid 41, Fever Typhus 2, Hip Disease 1, Inflammation of Bowels 3, Inflammation of Brain 1, Inflammation of Kidneys 1, Inflammation of Lungs 4, Inflammation of Peritoneum 5, Inflammation of Spine 1, Inflammation of Stomach 1, Intemperance 1, Marasmus 5, Measles 1, Old Age 3, Paralysis 1, Parturition 1, Premature Birth 1, Scrofula 1, Whooping Cough 14, Unknown 10. Total 171. Still-born 7. Under one year of age 46; between one and twenty years 67; between twenty and fifty years 38; over fifty 17; unknown 3; males 92; females 78; not given 1. Total, 171.

J. WHITTAKER, *Health Physician.*

